# O3.4. INCREASED ENGAGEMENT OF THE FRONTO-PARIETAL NETWORK AND DECREASED ENGAGEMENT OF THE DEFAULT-MODE – CINGULO-OPERCULAR – SENSORIMOTOR BETWEEN-NETWORK CONNECTIVITY IN FIRST-EPISODE PSYCHOSIS PATIENTS

**DOI:** 10.1093/schbul/sby015.202

**Published:** 2018-04-01

**Authors:** Eva Rikandi, Teemu Mäntylä, Tuula Kieseppä, Jaana Suvisaari, Tuukka Raij

**Affiliations:** 1 National Institute for Health and Welfare, Helsinki; 2 Helsinki University and Helsinki University Hospital; 3 Aalto University

## Abstract

**Background:**

The brain basis of psychotic disorders is still inadequately understood; however, evidence strongly suggests a central role of the dysfunctional integration of signaling between brain systems, i.e. “dysconnectivity”. Resting-state functional connectivity (FC) studies of chronic schizophrenia have revealed several illness-related, network-level changes. Patients have shown reduced modular structure, changes in the subcortical-cortical interactions, increased FC within the default-mode network (DMN) and reduced FC between fronto-parietal (FP) network components. Network-level changes in earlier stages of psychotic illnesses are less studied but DMN hyperconnectivity, loss of anticorrelation between task-positive and task-negative networks and both hypo- and hyper- corticostriatal connectivity have been reported in early and at-risk stages of psychosis. While studies using tasks and resting-state have yielded plenty of valuable information, regular fmri tasks capture only a narrow field of brain functioning and during rest, behavior may vary greatly between subjects. In this study we assessed subnetworks of first-episode psychosis (FEP) patients during processing of a movie stimulus that includes every-day-like rich variety of stimuli.

**Methods:**

We recorded 3T fMRI of 71 FEP patients and 57 control subjects, recruited from the Helsinki Early Psychosis Study, while they watched scenes from the movie Alice in Wonderland (Tim Burton, 2010). We then constructed a network of 160 nodes based upon a meta-analysis of regions related to a wide range of cognitive and emotional processing, we extracted signal time courses from each node and created a 160x160 correlation matrix for each subject. Using GraphVar software, we first identified all pairs of nodes where FC was statistically significantly different between groups (p < 0.05, FDR-corrected for multiple comparisons) and then extracted Graph-Components, or subnetworks, in which all pairs of nodes are connected by significant links.

**Results:**

We identified a statistically significant subnetwork of 49 nodes with mainly decreased but also some increased links of FC in patients. Nodes that had a high number of decreased FC links in patients were mostly situated bilaterally in the medial prefrontal (mPFC) regions of the DMN and subcortical regions of the cingulo-opercular (CO) network, concentrated in the basal ganglia. The decreased FC links of the DMN were relatively wide-spread, connecting to some nodes within the DMN and several nodes of the CO and sensorimotor (SM) networks as well as the cerebellum. The decreased FC links of the basal ganglia were mainly connected to nodes of the SM network and the cerebellum. Patients had nodes with several links of increased FC mainly in the FP network, connecting to nodes within the FP as well as nodes of the CO network and the cerebellum.

**Discussion:**

Our results indicate that during naturalistic stimulus, network-level changes in FC are already present at the early stages of psychoses implicating similar networks and links as seen in earlier studies using resting state and simple stimuli in mainly chronic patients. However, we found the within FP as well as FP-CO connectivity to be increased in patients, seemingly contradicting earlier evidence of reduced FC between FP components. It would seem that during movie viewing patients engage more regions involved in attentional control (perhaps for compensatory purposes) whereas control subjects have stronger involvement of regions related to spontaneous cognition and high-order integration.

